# Supplementary material for: Spectro-temporal vs. spectral features to predict the lombard gain in Mandarin Chinese
Source: PLoS One. 2026 Aug 26;21(8):e0356236. doi: 10.1371/journal.pone.0356236 (PMC13514036; doi:10.1371/journal.pone.0356236)
Supplement: Supplementary file 1 [file pone.0356236.s001.pdf]

Additional comments

2 Introduction

1. The discussion of intelligibility in non-tonal languages appears to overemphasize spectral cues. Temporal cues, including duration, speaking rate, and rhythm, are also known to contribute to intelligibility, particularly in noise.
2. In tonal languages such as Mandarin, F0 contours are typically considered suprasegmental rather than segmental. Referring to tone as a "segmental cue" may therefore be misleading, as segmental cues conventionally refer to consonant and vowel contrasts.

2.2 SI Models of this study

1. Claims that FADE outperforms ASR systems require greater specificity. The languages, noise conditions, and evaluation metrics (e.g., correlation with human SRTs, predicted SRT accuracy, word error rate) are not clearly defined. Without this context, references to outperforming "commercial ASR" may be misinterpreted as general ASR superiority rather than improved intelligibility prediction.
2. The rationale for why FADE performs poorly for absolute SRT but well for Lombard gain should be introduced earlier. Since LG is derived from absolute SRT, this contrast may appear inconsistent unless bias cancellation between speech styles is explicitly stated.

3.1 Empirical Data

1. The Lombard effect was elicited using 80 dB SPL noise over headphones. While plausible, this level is relatively high and raises questions about comfort, ecological validity, and variability in vocal effort. It is unclear whether noise levels were verified individually or whether speakers had prior Lombard-task exposure.
2. Lombard gain ranged from −0.6 to 3.2 dB. Although negative values are plausible, this wide range indicates substantial inter-speaker variability and suggests that Lombard speech is not uniformly beneficial. This should be acknowledged explicitly.

3.2.2 FADE Backend: The Generalized Optimum Detector

1. Although described as "spectral-only," FADE's HMM backend with eight states per word may still allow implicit temporal inference. This should be flagged as a limitation, as the system may not be fully insensitive to temporal structure.

4.1 Absolute SRT50 prediction

1. A clear discrepancy exists between absolute SRT and Lombard gain modeling. Spectral-only FADE showed poor fit for absolute SRT (χ^2/v up to 11.8, $p < 0.001$) but excellent fit for LG ($\chi^2/v \approx 1$, n.s.). Given that LG is derived from absolute SRTs, this contrast warrants explicit explanation, such as cancellation of systematic spectral biases when taking differences between speech styles.
2. Finally, speakers 10 and 11 appear to be outliers and may disproportionately influence correlation and χ^2 metrics; their impact is not discussed and should be considered.
