# Supplementary material for: Spectro-temporal vs. spectral features to predict the lombard gain in Mandarin Chinese
Source: PLoS One. 2026 Aug 26;21(8):e0356236. doi: 10.1371/journal.pone.0356236 (PMC13514036; doi:10.1371/journal.pone.0356236)
Supplement: Supplementary file 3 [file pone.0356236.s004.pdf]

Response to reviewers' Comments for the article "Spectro-temporal vs. Spectral Features to Predict the Lombard Gain in Mandarin Chinese"

July 24, 2026

Dear Dr. Wang,

We thank you and the two anonymous reviewers for the thoughtful comments in the second round of revisions of our manuscript. In the following, we provide our responses, highlighted in red, following the text of the letter. As before, a version of the revised manuscript with marked changes is appended to this document for your reference.

1 Review Comments of Reviewer #1

Thank you for addressing the previous comments. I believe the manuscript has improved substantially. I have a few remaining concerns that would benefit from additional clarification.

1.1

The proposed explanation that improved LG prediction accuracy arises from cancellation of a consistent bias between plain and Lombard SRT predictions is plausible, but it is currently not directly supported by evidence. Please clarify whether this bias has been empirically observed (e.g., via similar residual structure or error correlation across conditions), or alternatively frame this explanation more explicitly as a hypothesis or post hoc interpretation.

We thank the reviewer for pointing out that our previous explanation of this point was insufficient. We have revised the manuscript to clarify that any consistent bias in the predicted absolute SRTs of plain- and Lombard- speech would cancel out when computing the Lombard Gain (LG), since LG is defined as the difference between the two predicted SRTs. Consequently, a systematic bias affecting both conditions equally does not influence the LG predictions. By contrast, because the reported prediction performance for absolute SRTs is based on the RMS error (where $\mathrm{RMS}^2 \propto \mathrm{bias}^2$), a large systematic bias will result in poorer performance metrics in Tables 1 and 2. Therefore, poor prediction accuracy for the absolute SRTs does not necessarily imply poor prediction accuracy for the LG. The latter depends

primarily on the consistency of the relative prediction between the two conditions rather than on the absolute prediction bias. We reformulated the text in the manuscript: *Higher accuracy of lg predictions than of the absolute srt predictions is theoretically possible because any systematic prediction bias that affects both plain and Lombard speech similarly cancels when computing the lg prediction. Consequently, a large bias of the absolute srt prediction does not necessarily imply an equally bad performance of the lg prediction. While this mechanism provides a plausible explanation for the present results, the existence of such a shared prediction bias was not evaluated explicitly in this study.*

1.2

In addition, the robustness analysis excluding speakers 10 and 11 is currently described only qualitatively. Please clarify how these speakers were evaluated as non-outliers, specify the analysis performed, and provide quantitative evidence that the principal conclusions and model statistics remain stable when these speakers are excluded. Consider including formal influence diagnostics (e.g., Cook's distance or leverage) and/or a quantitative sensitivity analysis excluding these participants.

We now provide the results of the robustness analysis as supplementary material (Table 2). Specifically, we performed a quantitative sensitivity analysis by repeating the statistical analyses after excluding speaker 10 and 11. The results remained quantitatively consistent with those obtained from the full dataset, with all principal conclusions and statistical significance unchanged. This demonstrates that the inclusion/exclusion of speakers 10 and 11 does not influence the reported findings. We have added this clarification to the revised version of the manuscript. Rather than applying regression-based influence diagnostics such as Cook's distance, we performed a direct sensitivity analysis by repeating all statistical analyses after excluding the two speakers. We believe this approach is more appropriate because our conclusions are based on multiple prediction metrics rather than on a single regression model.

2 Review Comments of Reviewer #2

Thank you for the revision. Most of my concerns regarding temporal information have been addressed and it's clearer what it means in the context of this manuscript.

2.1

Given the more restricted definition of temporal information now, the research question is more like "whether explicitly encoding spectral-temporal modulation patterns (within each time frame) improves prediction of LG and SRT, compared to approaches that don't." The authors have clarified this in the methods (3.2.1) but I think the aim of the study (2.3) should also be revised accordingly to reflect this.

The key thing is to briefly explain why FADE with SGBFB features and with log-Mel spectrograms represent spectrotemporal vs. spectral-only approaches, so that readers would not

feel confused later by why log-Mel spectrograms are considered to lack temporal information.

We thank the reviewer for pointing out the missing changes in the section **2.3 Aim of this study and rationale**. We have added the necessary explanation.

2.2

Minor comments:

2.2.1

Section 2.1. Explain what α-ratio is.

We added a definition of the α-ratio in the section 2.1. Specifically, we state that the α-ratio is defined as the ratio of the speech energy between the frequency range 1 to 15 kHz and 50 to 1000 Hz.

2.2.2

P. 7, line 172: "temporal informatio" –> "information"

corrected.

Spectro-temporal vs. Spectral Features to Predict the Lombard Gain in Mandarin Chinese

Maximilian Karl Scharf[1], Anna Warzybok[1], Lena L.N. Wong[2], Fei Chen[3], Birger Kollmeier[1]

1 Medical Physics and Cluster of Excellence "Hearing4all", Carl von Ossietzky Universität Oldenburg, Germany.
2 Clinical Hearing Sciences (CHearS) Laboratory, Faculty of Education, The University of Hong Kong, Hong Kong SAR, China.
3 Department of Electrical and Electronic Engineering, Southern University of Science and Technology, Shenzhen, China.

* corresponding author:
maximilian.scharf@uni-oldenburg.de (MS)

1 Abstract

Background: In background noise, speakers adapt their speech production, giving rise to Lombard speech, which often improves speech intelligibility (SI). While intelligibility benefits of Lombard speech have been extensively studied in non-tonal languages, it remains unclear whether spectro-temporal cues, which are critical for tonal contrasts, are necessary to predict both the Lombard gain (LG) (i.e., intelligibility improvement relative to plain speech) and absolute SI in Mandarin Chinese.

Methods: Predictions of two SI-models were compared, namely, an automatic speech recognition (ASR)-based approach using spectral or spectro-temporal features and the speech intelligibility index (SII)-based model using spectral features. Predicted LG and absolute speech recognition threshold (SRT) values, for five female and six male speakers in stationary speech-shaped noise, were compared with empirical data.

Results: For both models, spectral features alone are sufficient for accurate prediction of the LG for both models. In contrast, predictions of absolute SRT were

most accurate when spectro-temporal features were included, capturing substantial inter-speaker variability.

Conclusions: Despite the tonal nature of Mandarin, spectro-temporal features are not required to predict the LG. However, they are essential to predict the absolute SRTs, which vary across speakers.

Keywords: speech intelligibility prediction, tonal language, Lombard effect

2 Introduction

Speech intelligibility (SI) depends on the acoustic cues that carry linguistic information in a given language. In non-tonal languages such as English or German, these cues are primarily determined by spectral properties of the speech signal. In tonal languages like Mandarin Chinese, however, dynamic changes of F0-contour within a single syllable carry lexical meaning and therefore constitute essential suprasegmental cues, i.e., cues within a single syllable. These tonal patterns reflect a distinct spectro-temporal structure, in addition to other temporal acoustic cues that are also present in non-tonal languages, such as duration, speaking rate, dynamic formant transitions, and F0-contours for prosodic categories. This raises the possibility that temporal dynamics play a distinct role for SI in tonal languages.

An additional factor influencing the acoustic realization of speech is the Lombard effect, which refers to the changes in a speaker's voice when speaking in loud acoustic environments [1]. The acoustic changes associated with the Lombard effect include an upward shift in fundamental frequency (F0), frequency shifts in the first and second formants (F1, F2), and a reduced speaking rate (see, for example, [2–7]), modifying both spectral and temporal properties of speech. These changes enhance audibility and clarity of speech cues, improving speech recognition [8–10].

The improvement in speech intelligibility between plain and Lombard speech is commonly referred to as the Lombard gain (LG). Spectral changes, such as changes in F0 and formants, strengthen the contrast of speech cues against masking sounds, while temporal changes, including a slower speaking rate, give listeners more opportunities to process the signal, particularly in fluctuating noises [11]. In contrast to absolute[1] SI, the

[1] By "absolute" SI prediction we denote a reference-free, ab-initio SI prediction as opposed to a

LG reflects a relative benefit and may therefore rely on a different subset of acoustic cues.

Although these mechanisms have been extensively studied in non-tonal languages, it remains unclear whether the same acoustic dimensions account for LG and absolute SI in tonal languages.

2.1 Tonal languages

For tonal languages, the Lombard effect may interact with the dynamic F0 patterns that carry lexical meaning. While spectral cues such as shifts in F0 and formants are likely important for the LG, the contribution of temporal information is still questionable.

Recent findings for Mandarin Chinese [7] suggest that spectral measures, such as α-ratio (ratio of the speech energy between 1 to 15 kHz and 50 to 1000 Hz) and speech-weighted signal to noise ratio (SNR), correlate with the LG, while F0-related changes are also predictive. However, the relative contributions of spectral versus spectro-temporal cues to absolute SI and LG in tonal languages remain unresolved.

To address this question, we explore computational SI models as a controlled analysis tool that allows us to isolate specific acoustic features. The models are not the primary object of investigation in this study; instead, they allow us to manipulate the available acoustic information selectively for a prediction, thereby disentangling spectral and spectro-temporal contributions under otherwise identical conditions.

2.2 SI models of this study

The primary model was the framework of auditory discrimination experiments (FADE) of [12]. FADE implements a machine learning backend, adapted from automatic speech recognition (ASR) systems, similar to the ”optimum detector” approach [13]. Stimuli are first preprocessed by a frontend, which is inspired by auditory-system mechanisms, allowing precise control over the type of information entering the model. This control makes it possible to directly compare speech recognition threshold (SRT) predictions using spectral or spectro-temporal representations of the input signals. Importantly, FADE does not assume any fixed or predefined combination of spectral and temporal

”relative” SI prediction of a deterioration or improvement in relation to a predefined standard.

cues; instead, it automatically selects the features that lead to the best speech recognition performance.

FADE has previously demonstrated high accuracy across several non-tonal languages in both stationary and fluctuating noise conditions [14], outperforming the extended speech intelligibility index (SII) (eSII, see [15]). It also provides accurate predictions for normal-hearing and hearing-impaired listeners [16, 17], and it still outperforms state-of-the-art commercial ASR systems [18] in the task of human SI prediction. In the context of tonal languages, FADE successfully predicted the average LG for Cantonese speakers [19], which further motivated its use in the present study.

Despite these advantages, the ASR backend of FADE is implemented as a hidden Markov model with restricted transitions (see method section). As a result, even a spectral-only input representation may still permit some spectro-temporal inference if its changes across time frames are decoded. Therefore, the classical SII was used as a second model approach, which decomposes speech and noise into long-term spectral components, providing a strictly spectral model of intelligibility [20].

The SII has proven to be a simple yet powerful SI model for non-tonal languages [21, 22]. If the LG in Mandarin Chinese is fully explainable by spectral changes in the speech signal only, a strictly spectral model such as the SII is expected to be sufficient to account for the changes in speech intelligibility.

2.3 Aim of this study and rationale

The presented study investigates whether spectral information alone is sufficient to predict the LG in Mandarin Chinese, or whether additional spectro-temporal cues are required. In addition, we examine whether the same conclusions hold for absolute SI, quantified by the SRT.

Because spectral features are a subset of spectro-temporal features, accurate predictions based solely on spectral inputs would imply that temporal cues introduced by the Lombard effect do not provide additional, independent intelligibility-relevant information. Under the assumption that human listeners make optimal use of the available cues, this would indicate that spectral changes are the main driving factor of the LG in Mandarin Chinese.

To address this, two complementary comparisons were carried out:

(1) FADE with spectro-temporal versus FADE with ~~spectral only~~ spectral-only features This comparison isolates the contribution of the input representation, as both conditions share the same computational backend. Any differences in predicted LG, therefore, stem solely from the presence or absence of temporal information in the frontend.

This is done by a comparison of the predictions with separable Gabor filter bank (SGBFB) features and log-mel features. Although both representations are extracted from time-varying speech signals, each log-mel feature vector contains only the spectral information of the current analysis frame, whereas SGBFB features explicitly encode spectro-temporal modulation patterns across adjacent frames (see Section 3.2.1). Consequently, within the present study, log-mel features are regarded as spectral-only information.

(2) FADE with spectral features versus SII This comparison ensures that predictions based on spectral information are evaluated with a strictly spectral model. As the models (i.e., FADE and SII) differ in their computational architectures, this step verifies that any effects observed in comparison (1) are not artifacts of residual temporal sensitivity in FADE's ASR-based backend.

Together, the two comparisons allow us to determine whether spectro-temporal cues provide indispensable information for predicting the LG, while also controlling for potential confounds arising from the underlying model architecture.

3 Methods

3.1 Empirical data

The empirical data of SRT of plain and Lombard speech in Mandarin were previously published by [7]. The speech material consisted of Mandarin matrix test sentences [23], a syntactically fixed, five-word sentence test with a constrained vocabulary. Recordings were obtained from six male and five female native Mandarin speakers for both plain and Lombard speech. To induce Lombard speech, unmodulated speech noise with a

long-term spectrum of a male speaker at normal vocal effort [24] (ICRA1) was presented to the speaker at a fixed level of 80 decibel (dB) sound pressure level (-SPL). This level was chosen based on previous studies, which showed that it reliably elicits Lombard speech while remaining below levels associated with hearing damage or excessive auditory or vocal fatigue [6]. The use of a fixed noise level was motivated by the aim of controlled elicitation of Lombard speech rather than ecological validity. The plain and Lombard speech recordings were subsequently used to measure speech recognition and assess the speaker-specific LG with a group of 13 native normal-hearing listeners. The SRT was measured adaptively with the procedure proposed by [25] in stationary ICRA1 noise. Twenty-two measurements were conducted with each listener so that the SRT was obtained for each speaker and speaking style (11 speakers x two speaking styles).

The average across listeners was used as speaker-specific SRT for each condition, and the uncertainty of the mean was estimated from the standard deviation across listeners. [7] reported speaker-specific SRTs in the range of -17.3 dB to -11.6 dB for plain speech and -18.7 dB to -12.4 dB for Lombard speech. The speaker-specific LGs varied from 3.2 dB to -0.6 dB, which indicated substantial inter-speaker variability. The smallest Lombard gain was on the same order as the average measurement uncertainty (≈ 0.5 dB). Despite this variability, the data showed a significant mean LG across speakers.

3.2 FADE prediction

[12] proposed the framework of auditory discrimination experiments (FADE) for SRT modeling. FADE uses a hidden Markov model with a Gaussian mixture model (HMM-GMM) backend adapted from ASR systems. This design allows the model to evaluate intelligibility from the acoustic input while retaining the flexibility to exploit spectral and temporal cues without any a priori assumptions about their combination.

3.2.1 FADE frontend: feature representation

FADE supports a wide variety of speech signal feature types for entry into the ASR backend. Because of the characteristics of the backend of the model (see section 3.2.2 below), only the representation within the individual time frames is accessible for the SI

prediction. Transitions between frames are not used as explicit speech features but instead contribute only to the state decoding of the underlying Markov model. Importantly, in this study, the term ”temporal” ~~informatio~~ information does not refer to the frame-wise sequence of spectral features, but rather to explicit spectro-temporal modulation patterns (e.g., as represented in the SGBFB filterbank frontend). Log-mel spectrogram features are therefore considered ”spectral” in the sense that they encode within-frame spectral energy distributions, while temporal dynamics are not explicitly represented as features. To test the contributions of spectral versus spectro-temporal cues to the Lombard gain in Mandarin Chinese, feature representations were selected that selectively encode information about the signal:

1. Log-mel spectrograms (31 bands, 16 kHz sampling rate, without differential, acceleration, or ~~higher order~~ higher-order coefficients [26]) primarily capture spectral content within each time frame, providing a baseline representation without explicit temporal modulations.

2. mel frequency cepstral coefficients (MFCC) without differential, acceleration, or ~~higher order~~ higher-order coefficients are derived from a downsampled inverse Fourier transform of the log-mel spectrogram. They also represent spectral information only, offering a complementary spectral-only baseline to verify that results are not dependent on a particular spectral representation.

3. SGBFB features apply a two-dimensional filter to the log-mel spectrogram, explicitly capturing both spectral and temporal modulations within each time frame. This representation is motivated by simple auditory principles, modeling cortical neuron responses and providing a means to incorporate the dynamic temporal patterns [27, 28] that may be especially relevant for tonal languages and Lombard speech.

By comparing model predictions across these feature sets, we can systematically evaluate the added value of temporal cues in the prediction of the LG in Mandarin Chinese, while controlling for differences in spectral representations.

3.2.2 FADE backend: the generalized optimum detector

Central to FADE is the concept of an optimum detector, which extracts the most relevant information from the input feature representation to perform the detection or discrimination task. Unlike the optimum detector approach of [29], FADE generalizes to

any psychoacoustic discrimination task, such as speech recognition, using a HMM-GMM as ASR backend. This flexibility makes FADE a suitable tool for testing the importance of cues that contribute to LG in Mandarin Chinese. In contrast, traditional SI-models, such as the SII, make fixed assumptions about the importance of signal features.

The optimum detector operates in two steps: in the first step, the speech signals of the test material (i.e., the sound files of the speaker for the condition of interest) are mixed with random segments of masking noise across a range of SNR. HMM-GMMs are then trained on the labeled mixtures for each SNR. The underlying Markov model assumes eight consecutive states per word, three states representing silence between words, and six states representing the onset or offset silence of a sentence. For the definition of the grammar in the language model, word transitions are restricted according to the grammar of the matrix test. Importantly, this is not an additional modeling assumption, but rather reflects the experimental condition of listeners who are trained before actual measurements to get familiar with the limited vocabulary of the test and with the sentence structure. Including the same grammar in FADE therefore ensures that the model operates under the same structural constraints as the human listeners. Accordingly, FADE models an optimally trained human listener.

In the second step of the optimum detector, a new set of data, constructed in the same manner but using new segments of noise, is used to test the performance of the HMM-GMMs. The resulting model performance as a function of training- and testing-SNR defines the predicted SRT: the lowest test SNR at which word recognition rate reached 50% is returned as the SRT. Uncertainties of the predictions are estimated by bootstrapping the word-recognition performance.

For SRT predictions, FADE is run independently for each speaker and speaking style (plain, Lombard), ensuring that the optimum detector is always trained on the specific material for which the prediction is made. The LG predictions are the difference between the predictions for plain and Lombard speech~~.~~:

:

$$\mathrm{LG} = \mathrm{SRT}_{\mathrm{plain}} - \mathrm{SRT}_{\mathrm{Lombard}} \tag{1}$$

Higher accuracy of LG predictions ~~in comparison to~~ than of the absolute SRT predictions ~~may be possible and is caused by cancellation of a consistent bias of~~ is

theoretically possible because any systematic prediction bias that affects both plain and Lombard speech similarly cancels when computing the LG prediction. Consequently, a large bias of the absolute SRT ~~predictions~~prediction does not necessarily imply an equally bad performance of the LG prediction. While this mechanism provides a plausible explanation for the present results, the existence of such a shared prediction bias was not evaluated explicitly in this study.

The Markov assumption of the FADE backend with its eight states per word warrants consideration when interpreting the predictions based on spectral features. This is because, although only spectral information may enter the backend, the Markov chain could, in principle, model limited temporal structure through state transitions across successive frames. However, this temporal modeling is strongly constrained by the small number of states per word, which prevents a detailed representation of fine-grained temporal dynamics. For example, an upwards shift or second tone in Mandarin over 500 ms with a window shift of 10 ms would require in the order of 50 states for log-mel features, exceeding the available state range. To address this limitation and ensure a comparison with a model that does not rely on any implicit temporal features, a purely spectral model (the SII, see following Section 3.3) was included in this study for comparison.

3.3 SII-based prediction

To complement the FADE predictions, which may be sensitive to limited temporal information, the speech intelligibility index (SII) [20] was used as a purely spectral model. Unlike FADE, the SII does not incorporate temporal modulations or spectro-temporal interactions and assumes that the filtered long-term spectrum fully determines SI.

In this study, third-octave band-pass filters were implemented according to [30] and used for the calculation of the SII. While these filters differ from the log-mel or MFCC features of FADE, they share the essential property that only long-term spectral information is available to the model.

3.3.1 Definition of the SII

For the SII calculation, speech and noise were decomposed into third-octave bands. The band-specific audibility was computed and weighted using Mandarin language- and gender-specific band importance functions (BIFs) [31].

3.3.2 Reference condition for SII-based predictions

To convert the SII into a predicted SRT, a reference SII ($\mathrm{SII}_{\mathrm{ref}}$) was calculated from empirically measured SRTs of the original Mandarin matrix test [23] in stationary ICRA1 noise. For a new speech in noise condition, the SNR is adjusted such that the SII matches the reference value:

$$\mathrm{SRT}_{\mathrm{pred}} = \underset{\mathrm{SNR}}{\mathrm{argmin}}\, |\mathrm{SII}_{\mathrm{ref}} - \mathrm{SII}(\mathrm{SNR})| \tag{2}$$

The SNR that corresponds to this match is taken as predicted SRT.

3.4 Statistical measures for a comparison of models

When comparing a model prediction with empirical data, it is important to account for measurement uncertainties and to understand how well a "perfect" model would perform.

Here, the "perfect" SI-model is defined as one that predicts the empirical SRT exactly when the measurement uncertainties are zero. In practice, both measurement and model predictions are associated with uncertainties, so even a perfect model deviates from the empirical data.

To quantify the agreement between model predictions and empirical data while considering these uncertainties, the chi-squared measure of goodness of a model (χ^2) was used:

$$\chi^2 = \sum_{i}^{\mathrm{N}} \frac{(a_i - b_i)^2}{\alpha_i^2 + \beta_i^2} \tag{3}$$

where a_i and b_i are the empirical and predicted values, respectively, while α_i and β_i are the standard deviations of measurement and prediction uncertainties. Good agreement of the prediction B with a measurement A is reflected by a value of χ^2 close

to the mean of the underlying χ^2-distribution. This is equal to the number of independently predicted b_i, also called the degrees of freedom ν.

A ratio χ^2/ν (also called normalized χ^2) close to one indicates that the model predictions deviate from empirical data by an amount consistent with the reported uncertainties. Values above one suggest that the model does not fully explain the data. $\chi^2/\nu \approx 0$ can only happen if measurement and prediction errors are correlated or overestimated.

The likelihood that the observed deviation arises purely from uncertainty can be evaluated using the χ^2-distribution. The probability $p_\nu\left(X > \chi^2\right)$ for the observed χ^2 is the likelihood that the mismatch between observation and prediction is the result of measurement errors and prediction uncertainties.

Commonly used statistical measures such as Pearson correlation coefficient (R), bias, and root mean square error (RMS) are included for comparability. In general, a good model should exhibit high R, low RMS, and bias and χ^2/ν in the range of unity.

4 Results

Table 1. Absolute SRT and LG prediction of empirical SRTs for Mandarin Chinese in ICRA1: Displayed are SII-based prediction and FADE predictions with different frontends. Pearson correlation coefficient (R), root mean square error (RMS), and bias are provided. χ^2/ν gives the normalized chi-squared measure. $p_\nu\left(X > \chi^2\right)$ gives the probability for χ^2 above or equal to the reported value for a χ^2-distribution with ν degrees of freedom. (See section 3.4)

	model	frontend	R	RMS/ dB	bias/ dB	χ^2/ν	ν	$p_\nu\left(X > \chi^2\right)$
abs. SRT	FADE	log-mel	0.94	1.35	1.15	4.6	22	<0.001
	FADE	MFCC	0.93	2.16	2.03	11.8	22	<0.001
	FADE	SGBFB	0.94	0.75	-0.003	1.7	22	0.021
	SII	-	0.89	1.32	-0.89	7.1	22	<0.001
LG	FADE	log-mel	0.87	0.48	0.05	0.8	11	0.640
	FADE	MFCC	0.78	0.72	0.34	1.7	11	0.067
	FADE	SGBFB	0.82	0.57	0.11	1.2	11	0.280
	SII	-	0.86	0.54	0.17	1.4	11	0.165

4.1 Absolute SRT50 prediction

Statistical comparisons of empirical and predicted SRTs are summarized in Table 1.

For absolute SRT predictions with the FADE model, the prediction accuracy strongly depended on the feature representation used. The best overall agreement with the empirical absolute SRTs was observed for FADE using SGBFB features with a high correlation (R=0.94), the lowest RMS error (0.75 dB), and negligible bias (−0.003 dB).

The normalized chi-squared was comparatively small ($\chi^2/\nu = 1.7$, $p_\nu = 0.021$), indicating that, although deviations remain statistically significant, the prediction errors were substantially closer to the expected uncertainty range than for any other model–feature combination. FADE using spectral features (log mel or MFCC) achieved similarly high correlations; however, the RMS error increased by a factor of about two for log-mel features and by almost a factor of three for MFCC features. Similarly, the bias increased markedly: predictions based on log-mel features showed a systematic offset of 1.15 dB, whereas the MFCC-based predictions exhibited an even larger bias of 2.03 dB. The normalized chi-squared values reflected these deviations, with $\chi^2/\nu = 4.6$ for log-mel and $\chi^2/\nu = 11.8$ for MFCC, indicating that both spectral feature sets failed to reproduce the empirical SRTs within the expected uncertainty range.

The SII performed less accurately than any FADE variant. Correlation was moderate (R=0.89), with an RMS error of 1.3 dB. The bias (−0.89 dB) was also noticeably larger in magnitude than for the FADE model with SGBFB. Most importantly, the normalized chi-squared remained high ($\chi^2/\nu = 7.1$, $p_\nu < 0.001$), indicating that the discrepancies between SII predictions and measured SRTs were larger than expected from the measurement uncertainty.

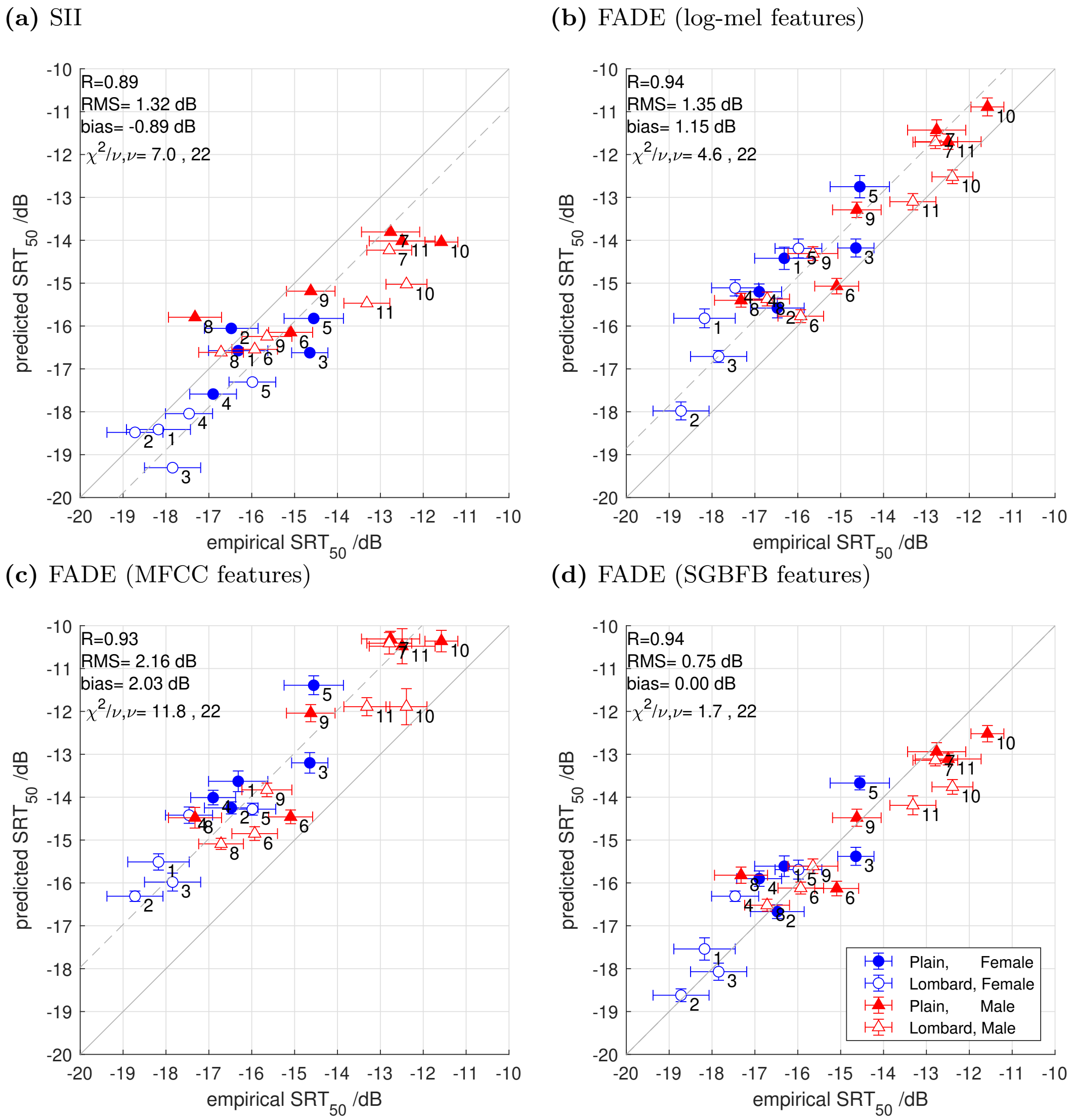

Fig 1. Absolute SRT prediction of Mandarin Chinese in ICRA1: Each speaker is assigned to a number. The dashed line represents a linear interpolation with a slope of unity. The bias is the offset between the dashed and continuous lines. Measurement- as well as model uncertainties (reported as standard deviation) are displayed as error bars. **1a:** SII-based prediction with a Mandarin female reference speaker from the original Mandarin Matrix sentence test of [23] with gender-specific BIF of [31].**1b** and **1c**: FADE prediction based on log-mel and MFCC features, respectively. These capture only spectral information.**1d:** FADE prediction based on SGBFB features, which encode spectro-temporal information within a single time-frame.

The measured and predicted SRTs, separated by speaker and speech style, are presented in Fig 1. Although both models produced SRTs that were generally too low for speakers 10 and 11, the remaining deviations appeared unsystematic, with no identifiable dependence on these speakers were not excluded from the primary analysis because there was no indication of systematic deviation associated with speech style or speaker gender. Instead, they were treated as valid observations representing the

natural variability of the dataset. To assess the influence of speakers 10 and 11 on the study conclusions, all statistical analyses were repeated after excluding these speakers (Table 2). The resulting performance metrics, statistical significance, and overall conclusions remained essentially unchanged, indicating that the reported findings are robust to their exclusion.

4.2 Lombard gain prediction

LG predictions are shown in Fig 2. Statistical comparisons are summarized in Table 1. FADE showed overall good agreement with the measured data. The log-mel features achieved the highest correlation ($R = 0.87$) together with the lowest error ($RMS = 0.48$ dB) and negligible bias (0.05 dB). The normalized chi-square value ($\chi^2/\nu = 0.8$,$p_\nu = 0.64$) indicated no significant difference between prediction mismatch and uncertainties.

FADE with SGBFB features, which was the best-performing configuration for absolute SRT predictions, also performed well for LG estimation. Its correlation ($R = 0.82$) and error ($RMS = 0.57$ dB) were slightly poorer than those obtained with log-mel features. The fit quality ($\chi^2/\nu = 1.2$, $p_\nu = 0.28$) likewise indicated no statistically significant deviation from the data. Despite the small differences across metrics, SGBFB features maintained a low bias (0.11 dB) and stable performance across tasks. FADE with MFCC features showed the weakest performance among FADE variants, reflected in lower correlation ($R = 0.78$), slightly increased prediction error ($RMS = 0.72$ dB). However, the chi-squared statistic ($\chi^2/\nu = 1.7$, $p_\nu = 0.067$) indicates that the mismatch is only slightly larger than expected from measurement and prediction uncertainties. Its bias was more pronounced (0.34 dB) but remained small in practical terms.

For comparison, the SII showed a similar correlation ($R = 0.86$) but a slightly higher error ($RMS = 0.54$ dB). Its bias remained small (0.17 dB), and the normalized chi-square value ($\chi^2/\nu = 1.4$, $p_\nu = 0.165$) indicated an acceptable, though less precise, fit than the best FADE variants.

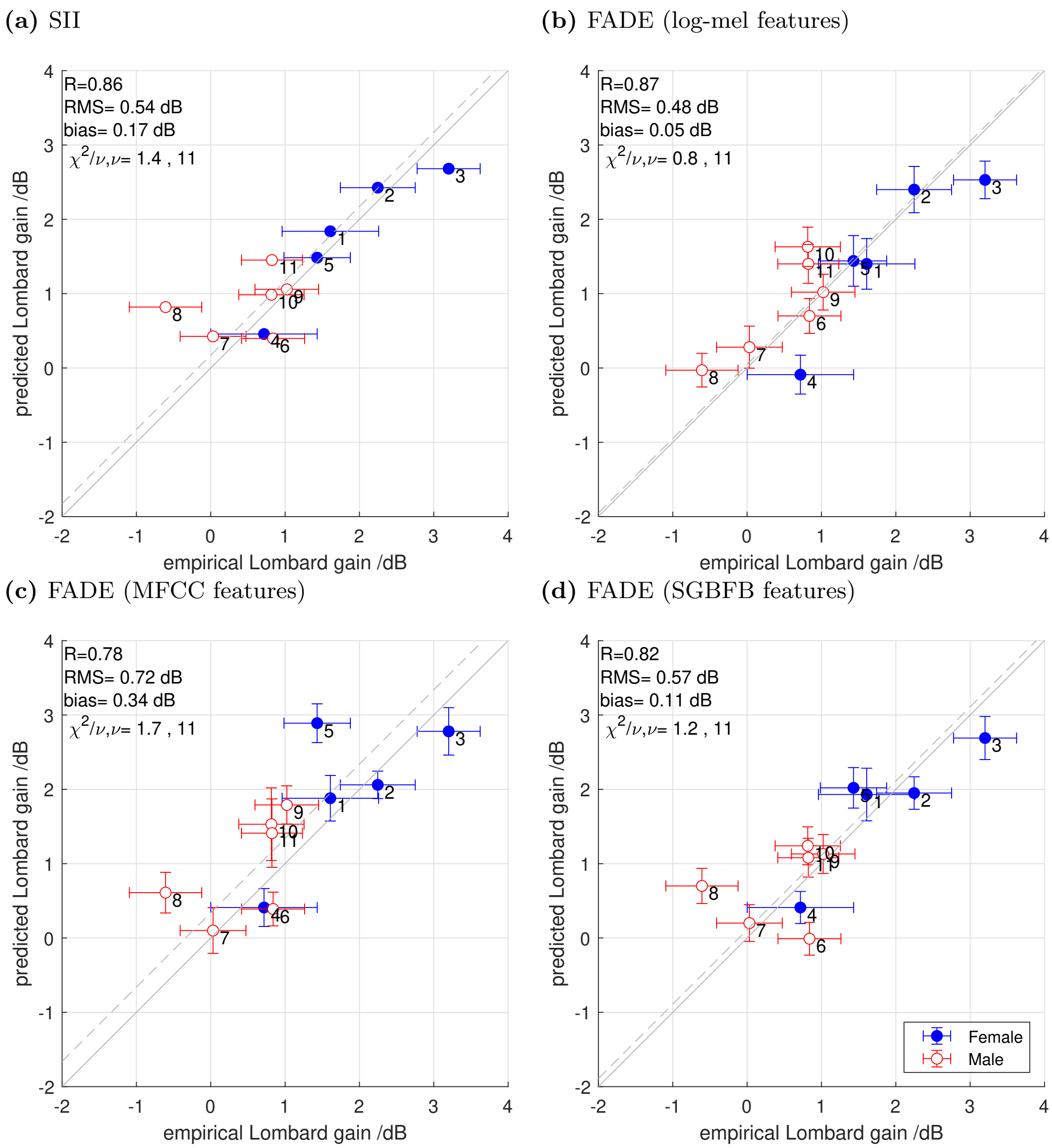

Fig 2. Lombard gain prediction of Mandarin Chinese in ICRA1: Each speaker is assigned to the same number as in Fig 1. The dashed line represents a linear interpolation with a slope of unity. The bias is the offset between the dashed and continuous lines. Measurement as well as prediction uncertainties (reported as standard deviation) are displayed as error bars. **2a:** SII-based prediction with a Mandarin female reference speaker from the original Mandarin Matrix sentence test of [23] with gender-specific BIF of [31]. **2b** and **2c**: FADE prediction based on log-mel and MFCC features, respectively. These capture only spectral information.**2d:** FADE prediction based on SGBFB features, which encode spectro-temporal information within a single time-frame. Overall, all approaches show equally accurate predictions.

5 Discussion

5.1 Absolute SRT50 prediction

The present results demonstrate that spectro-temporal features, i.e., spectro-temporal modulation patterns encoded within a single time-frame, are crucial for accurate, absolute SRT prediction. Speaker-specific SRTs can be predicted by both models; however, FADE with SGBFB features clearly outperformed all tested approaches. The SGBFB configuration of FADE showed the highest correlation with empirical data, the smallest RMS error, and no bias (−0.003 dB). Nevertheless, the measurement uncertainties were sufficiently small to reveal that neither FADE nor SII is a perfect model, as the remaining variance is higher than the expected variance from measurement uncertainties ($p_\nu \leq 0.021$ for all models; see Table 1). This indicates that factors beyond measurement uncertainties contribute to the mismatch and that more accurate models are theoretically possible.

One possible factor could be the limited frequency range of the FADE frontend, which sampled at 16kHz. This is supported by the findings of [32], who have shown that the frequency range above 8kHz significantly improves SI for normal hearing listeners. Improved SRT predictions within the FADE framework might be achieved by higher sampling rates in the frontend.

Nonetheless, the superior performance of FADE with SGBFB features for absolute SRT prediction suggests that encoding temporal information is essential for a speaker-specific SRT prediction, since only SGBFB capture dynamic spectro-temporal cues. Features such as individual time-frames of MFCC, and log-mel spectrograms, or the long-term spectrum used in the SII cannot encode these temporal aspects, which limits their predictive accuracy.

These findings align with [33], who demonstrated the advantage of gabor filter bank (GBFB) over MFCC in ASR tasks, highlighting the importance of spectro-temporal cues. Similar conclusions have been drawn across multiple languages, including German, Polish, Russian, Spanish, English, and Cantonese [14, 19], especially in fluctuating noise conditions [28]. Together, these results reinforce the role of spectro-temporal encoding as a key determinant of absolute SRT predictions for speech in stationary noise.

5.2 Lombard gain prediction

In contrast to absolute SRT predictions, the prediction of the LG relied primarily on spectral cues, i.e., cues within a single time-frame did not need to contain information about temporal modulations. FADE predicted the LG accurately even when using only spectral features (log-mel or the derived MFCC), with normalized chi-squared close to unity, indicating that the remaining mismatch could be fully explained by prediction- and measurement uncertainties.

FADE with SGBFB features, although superior for absolute SRT predictions, did not provide additional predictive value for the LG, suggesting that the temporal information is largely redundant for this measure. The SII showed comparable results, demonstrating that the information in the filtered long-term spectrum is sufficient to capture the relative improvements in SRT due to Lombard speech.

These findings are consistent with the acoustical properties of Lombard speech in Mandarin [7], where spectral properties of Lombard speech have been shown to correlate with the LG.

5.3 General remarks

This study examined the role of spectral and spectro-temporal[2] acoustic cues in predicting both absolute SI and the LG in Mandarin Chinese. As only a single tonal language was considered here and a comparison to non-tonal languages is beyond the scope of the current study, we cannot resolve whether tonal languages make use of spectro-temporal encoding differently from non-tonal languages. However, we found that the spectro-temporal features of speech are essential for accurate SRT prediction of Mandarin Chinese, similar to non-tonal languages [14]. This suggests that spectro-temporal representations are required to capture speaker-specific intelligibility differences, even when lexical tone is an inherent property of the language.

Previous work with bilingual speakers of English and Cantonese [19] supports the view that spectro-temporal features are not language-specific but rather encode the physical properties of speech production adequately, such as time-dependent spectral filtering due to articulatory movements in the vocal tract.

[2] i.e., spectral cues together with spectro-temporal modulation patterns encoded within a single time-frame.

In contrast, the prediction of the LG was equally accurate using FADE with either spectro-temporal or purely spectral features, as well as with the SII model. Within the measurement uncertainties of this study, spectro-temporal features did not provide additional predictive information beyond spectral cues for LG. This indicates that Lombard-induced intelligibility benefits in Mandarin are primarily driven by spectral changes, rather than by additional temporal information introduced by speaking rate or other dynamic modifications. At least in continuous ICRA1 noise, the Mandarin Chinese LG can be fully accounted for by spectral changes in the speech signal.

A limitation of this study is the size of the empirical dataset, which included 22 complete matrix tests from 13 listeners each. At the same time, it represents, to the authors' knowledge, the most extensive and most precise Mandarin plain/Lombard SRT dataset published. Nevertheless, this sample size may still be insufficient to detect subtle contributions of temporal cues to the LG, given the measurement uncertainty of approximately 0.5 dB. Accordingly, the absence of a spectro-temporal effect on LG should be interpreted as an upper bound on its potential contribution rather than definitive proof of its absence. Future studies with more listeners may clarify whether spectro-temporal features contain a small, previously undetectable influence on LG.

Similarly, although stationary, speech-shaped noise (ICRA1) was used here, other types of maskers, such as fluctuating or competing speech, may highlight temporal contributions that were not observed in the present study. The use of the Mandarin matrix test with monosyllabic and disyllabic words provides a controlled stimulus set. Still, future studies with everyday sentences or connected speech may uncover additional temporal effects, particularly those related to prosodic or contextual speech dynamics.

6 Conclusions

In summary, the main findings are:

1. Spectro-temporal speech features are essential for accurate absolute SRT prediction in Mandarin Chinese, capturing speaker-dependent differences in intelligibility.

2. Within the measurement uncertainty of this study, spectral features alone contain

all necessary information to predict the Lombard gain (LG) in continuous, speech-shaped noise (ICRA1) for Mandarin Chinese.

3. The speech intelligibility index (SII) and the framework of auditory discrimination experiments (FADE) are equally powerful models for predicting the LG. If only the LG, and not the absolute SRT, is of interest, the SII is preferable due to its lower complexity and faster calculations.

4. Future work using larger listener cohorts, additional noise types, and more realistic speech material is needed to determine whether subtle temporal contributions to LG emerge under less controlled conditions and whether these findings generalize across tonal and non-tonal languages.

7 Acknowledgements

7.1 Conflict statement

The authors declare that there is no conflict of interest.

7.2 Data availability

The FADE model is available under [34]. The speech test audio used in this study is available in [35]. The speech intelligibility (SI) data is available under [36].

7.3 Author contributions

MS was responsible for the simulation, analysis, and writing of the manuscript. AW, LW, FC, and BK were responsible for the data and funding. AW and BK contributed to the analysis and writing.

8 Supplementary Information

To test the influence of speakers 10 and 11 on the study conclusions, all statistical measures were repeated after excluding these speakers (see Table 2).

Table 2. Absolute SRT and LG prediction of empirical SRTs for Mandarin Chinese in ICRA1 without speakers 10 and 11: Displayed are SII-based prediction and FADE predictions with different frontends. (See Table 1)

	model	frontend	R	RMS/ dB	bias/ dB	χ^2/ν	ν	$p_\nu\left(X > \chi^2\right)$
abs. SRT	FADE	log-mel	0.92	1.47	1.32	5.4	18	<0.001
	FADE	MFCC	0.92	2.30	2.20	13	18	<0.001
	FADE	SGBFB	0.92	0.68	0.21	1.3	18	0.176
	SII	-	0.87	1.01	-0.60	3.5	18	<0.001
LG	FADE	log-mel	0.93	0.42	-0.09	0.51	9	0.868
	FADE	MFCC	0.79	0.73	0.27	1.9	9	0.047
	FADE	SGBFB	0.82	0.61	0.06	1.3	9	0.231
	SII	-	0.87	0.56	0.12	1.4	9	0.182

Acronyms

χ^2

chi-squared measure of goodness of a model

-SPL

sound pressure level

dB

decibel

ASR

automatic speech recognition

BIF

band importance function

FADE

framework of auditory discrimination experiments

GBFB

gabor filter bank

HMM-GMM

hidden Markov model with a Gaussian mixture model

ICRA1

unmodulated speech noise with a long-term spectrum of a male speaker at normal vocal effort [24]

LG

Lombard gain

MFCC

mel frequency cepstral coefficients

R

Pearson correlation coefficient

RMS

root mean square error

SGBFB

separable Gabor filter bank

SI

speech intelligibility

SII

speech intelligibility index

SNR

signal to noise ratio

SRT

speech recognition threshold

References

1. Lombard E. Le signe de l'elevation de la voix. Ann Mal de L'Oreille et du Larynx. 1911:101-19.

2. Hanley TD, Steer MD. Effect of Level of Distracting Noise upon Speaking Rate, Duration and Intensity. Journal of Speech and Hearing Disorders. 1949 Dec;14(4):363-8. doi:10.1044/jshd.1404.363.

3. Hansen JHL. Analysis and compensation of speech under stress and noise for environmental robustness in speech recognition. Speech Communication. 1996 Nov;20(1-2):151-73. doi:10.1016/s0167-6393(96)00050-7.

4. Pittman AL, Wiley TL. Recognition of Speech Produced in Noise. Journal of Speech, Language, and Hearing Research. 2001 Jun;44(3):487-96. doi:10.1044/1092-4388(2001/038).

5. Lu Y, Cooke M. Speech production modifications produced by competing talkers, babble, and stationary noise. The Journal of the Acoustical Society of America. 2008 Nov;124(5):3261-75. doi:10.1121/1.2990705.

6. Alghamdi N, Maddock S, Marxer R, Barker J, Brown GJ. A corpus of audio-visual Lombard speech with frontal and profile views. Journal of the Acoustical Society of America. 2018;143(6):523-9. doi:10.1121/1.5042758.

7. Chen F, Pan C, Hu H, Hochmuth S, Kollmeier B, Warzybok A. Understanding the Lombard Effect for Mandarin: Relation Between Speech Recognition Thresholds and Acoustic Parameters. Trends in Hearing. 2025 Mar;29. doi:10.1177/23312165251324266.

8. Dreher JJ, O'Neill J. Effects of Ambient Noise on Speaker Intelligibility for Words and Phrases. The Journal of the Acoustical Society of America. 1957 Dec;29(12):1320-3. doi:10.1121/1.1908780.

9. Summers WV, Pisoni DB, Bernacki RH, Pedlow RI, Stokes MA. Effects of noise on speech production: Acoustic and perceptual analyses. The Journal of the Acoustical Society of America. 1988 Sep;84(3):917-28. doi:10.1121/1.396660.

10. Junqua JC. The Lombard reflex and its role on human listeners and automatic speech recognizers. Journal of the Acoustical Society of America. 1993;93(1):510-24. doi:10.1121/1.405631.

11. Lu Y, Cooke M. The contribution of changes in F0 and spectral tilt to increased intelligibility of speech produced in noise. Speech Communication. 2009 Dec;51(12):1253-62. doi:10.1016/j.specom.2009.07.002.

12. Schädler MR, Warzybok A, Hochmuth S, Kollmeier B. Matrix sentence intelligibility prediction using an automatic speech recognition system. International Journal of Audiology. 2015 May;54(sup2):100-7. doi:10.3109/14992027.2015.1061708.

13. Dau T, Kollmeier B, Kohlrausch A. A quantitative prediction of modulation masking with an optimal-detector model. The Journal of the Acoustical Society of America. 1996 Apr;99(4):2565-74. doi:10.1121/1.415040.

14. Schädler MR, Warzybok A, Ewert SD, Kollmeier B. A simulation framework for auditory discrimination experiments: Revealing the importance of across-frequency processing in speech perception. Journal of the Acoustical Society of America. 2016;139(5):2708-22. doi:10.1121/1.4948772.

15. Rhebergen KS, Versfeld NJ, Dreschler WA. Extended speech intelligibility index for the prediction of the speech reception threshold in fluctuating noise. Journal of the Acoustical Society of America. 2006;120(6):3988-97. doi:10.1121/1.2358008.

16. Schädler MR, Hülsmeier D, Warzybok A, Kollmeier B. Individual Aided Speech-Recognition Performance and Predictions of Benefit for Listeners With Impaired Hearing Employing FADE. Trends in Hearing. 2020;24:233121652093892. doi:10.1177/2331216520938929.

17. Hülsmeier D, Warzybok A, Kollmeier B, Schädler MR. Simulations with FADE of the effect of impaired hearing on speech recognition performance cast doubt on the role of spectral resolution. Hearing Research. 2020 Sep;395:107995. doi:10.1016/j.heares.2020.107995.

18. Polspoel S, Moore DR, Swanepoel DW, Kramer SE, Smits C. Automatic development of speech-in-noise hearing tests using machine learning. Scientific Reports. 2025 Apr;15(1). doi:10.1038/s41598-025-96312-z.

19. Scharf MK, Hochmuth S, Wong LN, Kollmeier B, Warzybok A. Lombard Effect for Bilingual Speakers in Cantonese and English: importance of spectro-temporal features. In: Ko H, Hansen JHL, editors. Proc. Interspeech 2022. Incheon, South Korea: ISCA; 2022. doi:10.21437/Interspeech.2022-10235.

20. 5 A. Methods for calculating the speech intelligibility index SII. American National Standard; 1997.

21. Beutelmann R, Brand T, Kollmeier B. Revision, extension, and evaluation of a binaural speech intelligibility model. Journal of the Acoustical Society of America. 2010;127(4):2479-97. doi:10.1121/1.3295575.

22. Lopez-Poveda EA, Johannesen PT, Pérez-González P, Blanco JL, Kalluri S, Edwards B. Predictors of Hearing-Aid Outcomes. Trends in Hearing. 2017;21:233121651773052. doi:10.1177/2331216517730526.

23. Hu H, Xi X, Wong LLN, Hochmuth S, Warzybok A, Kollmeier B. Construction and evaluation of the Mandarin Chinese matrix (CMNmatrix) sentence test for the assessment of speech recognition in noise. International Journal of Audiology. 2018 Sep;57(11):838-50. doi:10.1080/14992027.2018.1483083.

24. Dreschler WA, Verschuure H, Ludvigsen C, Westermann S. ICRA Noises: Artificial Noise Signals with Speech-like Spectral and Temporal Properties for Hearing Instrument Assessment: Ruidos ICRA: Señates de ruido artificial con espectro similar al habla y propiedades temporales para pruebas de instrumentos auditivos. International Journal of Audiology. 2001 Jan;40(3):148-57. doi:10.3109/00206090109073110.

25. Brand T, Kollmeier B. Efficient adaptive procedures for threshold and concurrent slope estimates for psychophysics and speech intelligibility tests. Journal of the Acoustical Society of America. 2002;111(6):2801-10. doi:10.1121/1.1479152.

26. ETSI. Speech processing, transmission and quality aspects (STQ); distributed speech recognition; frontend feature extraction algorithm; compression algorithms. ETSI ES. 2003;201(108):V1.

27. Schädler MR, Kollmeier B. Separable spectro-temporal Gabor filter bank features: Reducing the complexity of robust features for automatic speech recognition. Journal of the Acoustical Society of America. 2015 Apr;137(4):2047-59. doi:10.1121/1.4916618.

28. Schädler MR, Warzybok A, Ewert SD, Kollmeier B. Robust automatic speech recognition and modeling of auditory discrimination experiments with auditory spectro-temporal features [PhD thesis]. Carl von Ossietzky Universität Oldenburg; 2016. Available from: `http://oops.uni-oldenburg.de/2844/`.

29. Dau T, Püschel D, Kohlrausch A. A quantitative model of the "effective" signal processing in the auditory system. I. Model structure. The Journal of the Acoustical Society of America. 1996 Jun;99(6):3615-22. doi:10.1121/1.414959.

30. 11 A. Specification for Octave, Half-Octave, and Third Octave Band Filter Sets. American National Standard; 2004. Available from: `https://archive.org/details/gov.law.ansi.s1.11.2004/`.

31. Chen J, Huang Q, Wu X. Frequency importance function of the speech intelligibility index for Mandarin Chinese. Speech Communication. 2016 Oct;83:94-103. doi:10.1016/j.specom.2016.07.009.

32. Polspoel S, Kramer SE, van Dijk B, Smits C. The Importance of Extended High-Frequency Speech Information in the Recognition of Digits, Words, and Sentences in Quiet and Noise. Ear & Hearing. 2021 Nov;43(3):913-20. doi:10.1097/aud.0000000000001142.

33. Schädler MR, Meyer BT, Kollmeier B. Spectro-temporal modulation subspace-spanning filter bank features for robust automatic speech recognition. The Journal of the Acoustical Society of America. 2012 May;131(5):4134-51. doi:10.1121/1.3699200.

34. Schädler MR, Hülsmeier D, Grimm G. m-r-s/fade: FADE version 2.4.1. Zenodo; 2021. Available from: https://zenodo.org/record/4730596. doi:10.5281/ZENODO.3734164.

35. Hu H, Warzybok A, Scharf M, Kollmeier B, Chen F, Hochmuth S. Mandarin matrix recordings: Lombard and plain speech with different speakers. Zenodo; 2022. doi:10.5281/ZENODO.7063030.

36. Chen F, Pan C, Hu H, Hochmuth S, Kollmeier B, Scharf MK, et al.. Mandarin matrix sentence test speech recognition thresholds (SRTs): Lombard and plain speech with different speakers. Zenodo; 2026. doi:10.5281/ZENODO.19110092.
