# Supplementary material for: Spectro-temporal vs. spectral features to predict the lombard gain in Mandarin Chinese
Source: PLoS One. 2026 Aug 26;21(8):e0356236. doi: 10.1371/journal.pone.0356236 (PMC13514036; doi:10.1371/journal.pone.0356236)
Supplement: Peer review history

# Peer review: Decision Letter 0

**DOI:** 10.1371/journal.pone.0356236.r001

**Authors:** Jie Wang

3 Mar 2026

Dear Dr. Scharf,

Thank you for submitting your manuscript to PLOS ONE. After careful consideration, we feel that it has merit but does not fully meet PLOS ONE’s publication criteria as it currently stands. Therefore, we invite you to submit a revised version of the manuscript that addresses the points raised during the review process.

Please submit your revised manuscript by Apr 17 2026 11:59PM. If you will need more time than this to complete your revisions, please reply to this message or contact the journal office at plosone@plos.org. When you're ready to submit your revision, log on to https://www.editorialmanager.com/pone/ and select the 'Submissions Needing Revision' folder to locate your manuscript file.

- A letter that responds to each point raised by the academic editor and reviewer(s). You should upload this letter as a separate file labeled 'Response to Reviewers'.
- A marked-up copy of your manuscript that highlights changes made to the original version. You should upload this as a separate file labeled 'Revised Manuscript with Track Changes'.
- An unmarked version of your revised paper without tracked changes. You should upload this as a separate file labeled 'Manuscript'.

If you would like to make changes to your financial disclosure, please include your updated statement in your cover letter. Guidelines for resubmitting your figure files are available below the reviewer comments at the end of this letter.

If applicable, we recommend that you deposit your laboratory protocols in protocols.io to enhance the reproducibility of your results. Protocols.io assigns your protocol its own identifier (DOI) so that it can be cited independently in the future. For instructions see: https://journals.plos.org/plosone/s/submission-guidelines#loc-laboratory-protocols. Additionally, PLOS ONE offers an option for publishing peer-reviewed Lab Protocol articles, which describe protocols hosted on protocols.io. Read more information on sharing protocols at https://plos.org/protocols?utm_medium=editorial-email&utm_source=authorletters&utm_campaign=protocols.

We look forward to receiving your revised manuscript.

Kind regards,

Jie Wang, Ph.D.

Academic Editor

PLOS One

Journal Requirements:

When submitting your revision, we need you to address these additional requirements.

1. Please ensure that your manuscript meets PLOS ONE's style requirements, including those for file naming. The PLOS ONE style templates can be found at

https://journals.plos.org/plosone/s/file?id=wjVg/PLOSOne_formatting_sample_main_body.pdf and

https://journals.plos.org/plosone/s/file?id=ba62/PLOSOne_formatting_sample_title_authors_affiliations.pdf

2. Please update your submission to use the PLOS LaTeX template. The template and more information on our requirements for LaTeX submissions can be found at http://journals.plos.org/plosone/s/latex.

3. Thank you for stating the following financial disclosure:

“This work was funded by: the Deutsche Forschungsgemeinschaft (DFG, German Research Foundation) - Project ID 415895050 - ''Experiments and models of speech recognition across tonal and non-tonal language systems (EMSATON)'', the Deutsche Forschungsgemeinschaft (DFG, German Research Foundation) under Germany’s Excellence Strategy – EXC 2177/1 - Project ID 390895286 - ''Hearing4all'' and the Deutsche Forschungsgemeinschaft (DFG, German Research Foundation) – Project ID 352015383 – ''SFB 1330 A5 Model-based diagnostics and hearing aid fitting in complex acoustic scenarios: Perceptive Principles, Algorithms and Applications'', the Deutsche Forschungsgemeinschaft (DFG) - Project ID 465121786 (GRUSTAD)”

Please state what role the funders took in the study.  If the funders had no role, please state: "The funders had no role in study design, data collection and analysis, decision to publish, or preparation of the manuscript." If this statement is not correct you must amend it as needed.

Please include this amended Role of Funder statement in your cover letter; we will change the online submission form on your behalf.

4. Please note that funding information should not appear in the Acknowledgments section or other areas of your manuscript. We will only publish funding information present in the Funding Statement section of the online submission form. Please remove any funding-related text from the manuscript.

5. Please note that your Data Availability Statement is currently missing the repository name and a direct link to access each database. If your manuscript is accepted for publication, you will be asked to provide these details on a very short timeline. We therefore suggest that you provide this information now, though we will not hold up the peer review process if you are unable.

6. Please ensure that you refer to Figure 2 in your text as, if accepted, production will need this reference to link the reader to the figure.

7. If the reviewer comments include a recommendation to cite specific previously published works, please review and evaluate these publications to determine whether they are relevant and should be cited. There is no requirement to cite these works unless the editor has indicated otherwise.

**Additional Editor Comments:**

The authors need to address the comments raised by the two reviewers.

[Note: HTML markup is below. Please do not edit.]

Reviewers' comments:

Reviewer's Responses to Questions

**Comments to the Author**

1. Is the manuscript technically sound, and do the data support the conclusions?

Reviewer #1: Yes

Reviewer #2: Yes

**********

2. Has the statistical analysis been performed appropriately and rigorously?

Reviewer #1: Yes

Reviewer #2: Yes

**********

3. Have the authors made all data underlying the findings in their manuscript fully available?

The PLOS Data policy

Reviewer #1: Yes

Reviewer #2: Yes

**********

4. Is the manuscript presented in an intelligible fashion and written in standard English?

Reviewer #1: Yes

Reviewer #2: Yes

**********

Reviewer #1: The study is strong and clearly of publishable quality. The experimental design is sound, the analyses are thorough, and the work makes a valuable contribution to the intelligibility modeling literature. That said, several conceptual and interpretive claims would benefit from clarification or more cautious wording to avoid potential misinterpretation by readers. I view these points as refinements rather than fundamental concerns, and I believe they can be addressed without major changes to the study. I look forward to following this line of work as it continues to develop. Please see attached comments.

Reviewer #2: This study evaluates whether spectral or spectro-temporal acoustic information is required to predict speech intelligibility in Mandarin Chinese. Two models were compared: an ASR-based FADE model implemented with either spectral or spectro-temporal features, and a speech intelligibility index (SII) model relying solely on spectral information. Model predictions of absolute speech recognition thresholds (SRTs) and Lombard gain (LG) were compared with previously collected behavioral data. Results showed that LG could be predicted accurately using spectral features alone, whereas accurate prediction of absolute SRT required spectro-temporal features in the ASR model. This indicated that temporal cues are important for modeling absolute intelligibility but not relative Lombard benefit in Mandarin.

The manuscript is clearly written and technically sound. The proposed ASR-based approach has the potential to provide a more flexible and accurate estimate of intelligibility than the traditional SII model. However, I recommend a revision to clarify some key terms and concepts before the manuscript is suitable for publication.

My major comment concerns what constitutes “temporal” properties in this study. The motivation for examining Mandarin is that tonal patterns are described as having an inherently spectro-temporal structure that may make temporal dynamics more important for intelligibility (p. 2, lines 38-40). However, this appears to imply that non-tonal languages such as English or German contain less temporal structure. I don’t think this assumption is well justified. Non-tonal languages also rely heavily on time-varying acoustic cues, including dynamic formant transitions for segmental identity and F0 contours for prosodic categories (e.g., pitch accents) crucial for intonational meaning. It would therefore be helpful for the authors to clarify in what sense tonal languages are expected to be “more temporal” than non-tonal languages, or to provide supporting evidence for this distinction.

Relatedly, I am also puzzled as to why the log-mel spectrogram features in the FADE frontend are described as “spectral-only.” A spectrogram has multiple time frames and therefore preserves temporal variation across frames. It appears that, in this manuscript, “temporal” specifically refers to explicit spectro-temporal modulation patterns captured by the separable Gabor filterbank representation rather than temporal variation per se. This should be stated explicitly, and the authors should provide a clearer definition of what they mean by “temporal” features throughout the manuscript.

Given the ambiguity in how temporal information is defined here, the authors should be more cautious in suggesting that “temporal” cues contribute more strongly to speech intelligibility in tonal languages when motivating the study. In fact, I think the present findings (e.g., FADE model with SGBFB best predicted absolute SRT) may generalize to non-tonal languages as well, although, as noted in the General Remarks, this would need to be empirically tested.

Another minor question concerns the frequency range represented in the FADE and SII models. The use of log-mel spectrograms with a 16 kHz sampling rate (line 156) implies that all feature representations are limited to frequencies below 8 kHz. Indeed, the SII model assigns low importance to frequencies above 8 kHz, but there is accumulating evidence suggests that extended high-frequency information (>8 kHz) can improve speech recognition performance in both (Polspoel et al., 2022; Motlagh Zadeh et al., 2019; Monson et al., 2019) and ASR (Guo & Chandrasekaran, 2025). The authors may wish to comment on this limitation and discuss whether incorporating higher-frequency information could further improve the predictive accuracy of the FADE model.

Other comments:

1. The χ²/ν metric in the abstract is difficult to interpret without context. Consider either removing it or briefly explaining its meaning.

2. P. 7, lines 185-187: “The underlying Markov model assumes eight consecutive states per word (three representing silence and six states representing…).” Is “eight” a typo? The numbers numbers appear inconsistent (3 + 6 is not 8).

3. Formula (1) on p. 9: Should the notation use absolute value bars (| |) rather than brackets [ ], as the goal here was to find the SNR that minimized the difference between SII and SII_ref?

References:

Polspoel, S., Kramer, S. E., van Dijk, B., & Smits, C. (2022). The importance of extended high-frequency speech information in the recognition of digits, words, and sentences in quiet and noise. Ear and hearing, 43(3), 913-920.

Motlagh Zadeh, L., Silbert, N. H., Sternasty, K., Swanepoel, D. W., Hunter, L. L., & Moore, D. R. (2019). Extended high-frequency hearing enhances speech perception in noise. Proceedings of the National Academy of Sciences, 116(47), 23753-23759.

Monson, B. B., Rock, J., Schulz, A., Hoffman, E., & Buss, E. (2019). Ecological cocktail party listening reveals the utility of extended high-frequency hearing. Hearing Research, 381, 107773.

Guo, Z. C., & Chandrasekaran, B. (2025). Extended high frequencies improve phoneme recognition: Evidence from automatic speech recognition in spatial speech mixtures. The Journal of the Acoustical Society of America, 158(4), 3365-3377.

**********

what does this mean?). If published, this will include your full peer review and any attached files.

If you choose “no”, your identity will remain anonymous but your review may still be made public.

**Do you want your identity to be public for this peer review?**  For information about this choice, including consent withdrawal, please see our Privacy Policy

Reviewer #1: No

Reviewer #2: No

**********

[NOTE: If reviewer comments were submitted as an attachment file, they will be attached to this email and accessible via the submission site. Please log into your account, locate the manuscript record, and check for the action link "View Attachments". If this link does not appear, there are no attachment files.]

To ensure your figures meet our technical requirements, please review our figure guidelines: https://journals.plos.org/plosone/s/figures

You may also use PLOS’s free figure tool, NAAS, to help you prepare publication quality figures: https://journals.plos.org/plosone/s/figures#loc-tools-for-figure-preparation.

NAAS will assess whether your figures meet our technical requirements by comparing each figure against our figure specifications.

Attachment Submitted filename: PONE_comments.pdf

# Author comment: Author response to Decision Letter 1

**DOI:** 10.1371/journal.pone.0356236.r002

11 May 2026

See pdf: Response to Reviewers.

Attachment Submitted filename: LombardGainModel_PLOSONE_rebuttalLetter.pdf

# Peer review: Decision Letter 1

**DOI:** 10.1371/journal.pone.0356236.r003

**Authors:** Jie Wang

19 May 2026

Dear Dr. Scharf,

Thank you for submitting your manuscript to PLOS ONE. After careful consideration, we feel that it has merit but does not fully meet PLOS ONE’s publication criteria as it currently stands. Therefore, we invite you to submit a revised version of the manuscript that addresses the points raised during the review process.

Please submit your revised manuscript by Jul 03 2026 11:59PM. If you will need more time than this to complete your revisions, please reply to this message or contact the journal office at plosone@plos.org. When you're ready to submit your revision, log on to https://www.editorialmanager.com/pone/ and select the 'Submissions Needing Revision' folder to locate your manuscript file.

- A letter that responds to each point raised by the academic editor and reviewer(s). You should upload this letter as a separate file labeled 'Response to Reviewers'.
- A marked-up copy of your manuscript that highlights changes made to the original version. You should upload this as a separate file labeled 'Revised Manuscript with Track Changes'.
- An unmarked version of your revised paper without tracked changes. You should upload this as a separate file labeled 'Manuscript'.

If you would like to make changes to your financial disclosure, please include your updated statement in your cover letter. Guidelines for resubmitting your figure files are available below the reviewer comments at the end of this letter.

If applicable, we recommend that you deposit your laboratory protocols in protocols.io to enhance the reproducibility of your results. Protocols.io assigns your protocol its own identifier (DOI) so that it can be cited independently in the future. For instructions see: https://journals.plos.org/plosone/s/submission-guidelines#loc-laboratory-protocols. Additionally, PLOS ONE offers an option for publishing peer-reviewed Lab Protocol articles, which describe protocols hosted on protocols.io. Read more information on sharing protocols at https://plos.org/protocols?utm_medium=editorial-email&utm_source=authorletters&utm_campaign=protocols.

As the corresponding author, your ORCID iD is verified in the submission system and will appear in the published article. PLOS supports the use of ORCID, and we encourage all coauthors to register for an ORCID iD and use it as well. Please encourage your coauthors to verify their ORCID iD within the submission system before final acceptance, as unverified ORCID iDs will not appear in the published article. *Only*  the individual author can complete the verification step; PLOS staff *cannot*  verify ORCID iDs on behalf of authors.

We look forward to receiving your revised manuscript.

Kind regards,

Jie Wang, Ph.D.

Academic Editor

PLOS One

Journal Requirements:

If the reviewer comments include a recommendation to cite specific previously published works, please review and evaluate these publications to determine whether they are relevant and should be cited. There is no requirement to cite these works unless the editor has indicated otherwise.

Please review your reference list to ensure that it is complete and correct. If you have cited papers that have been retracted, please include the rationale for doing so in the manuscript text, or remove these references and replace them with relevant current references. Any changes to the reference list should be mentioned in the rebuttal letter that accompanies your revised manuscript. If you need to cite a retracted article, indicate the article’s retracted status in the References list and also include a citation and full reference for the retraction notice.

Additional Editor Comments:

The authors need to address the comments of the two reviewers.

[Note: HTML markup is below. Please do not edit.]

Reviewers' comments:

Reviewer's Responses to Questions

**Comments to the Author**

Reviewer #1: (No Response)

Reviewer #2: All comments have been addressed

**********

2. Is the manuscript technically sound, and do the data support the conclusions?

Reviewer #1: Yes

Reviewer #2: Yes

**********

3. Has the statistical analysis been performed appropriately and rigorously?

Reviewer #1: Yes

Reviewer #2: Yes

**********

4. Have the authors made all data underlying the findings in their manuscript fully available?

The PLOS Data policy

Reviewer #1: Yes

Reviewer #2: Yes

**********

5. Is the manuscript presented in an intelligible fashion and written in standard English?

Reviewer #1: Yes

Reviewer #2: Yes

**********

Reviewer #1: Thank you for addressing the previous comments. I believe the manuscript has improved substantially. I have a few remaining concerns that would benefit from additional clarification.

The proposed explanation that improved LG prediction accuracy arises from cancellation of a consistent bias between plain and Lombard SRT predictions is plausible, but it is currently not directly supported by evidence. Please clarify whether this bias has been empirically observed (e.g., via similar residual structure or error correlation across conditions), or alternatively frame this explanation more explicitly as a hypothesis or post hoc interpretation.

In addition, the robustness analysis excluding speakers 10 and 11 is currently described only qualitatively. Please clarify how these speakers were evaluated as non-outliers, specify the analysis performed, and provide quantitative evidence that the principal conclusions and model statistics remain stable when these speakers are excluded. Consider including formal influence diagnostics (e.g., Cook’s distance or leverage) and/or a quantitative sensitivity analysis excluding these participants.

Reviewer #2: Thank you for the revision. Most of my concerns regarding temporal information have been addressed and it’s clearer what it means in the context of this manuscript. Given the more restricted definition of temporal information now, the research question is more like “whether explicitly encoding spectral-temporal modulation patterns (within each time frame) improves prediction of LG and SRT, compared to approaches that don’t.” The authors have clarified this in the methods (3.2.1) but I think the aim of the study (2.3) should also be revised accordingly to reflect this. The key thing is to briefly explain why FADE with SGBFB features and with log-Mel spectrograms represent spectrotemporal vs. spectral-only approaches, so that readers would not feel confused later by why log-Mel spectrograms are considered to lack temporal information.

Minor comments:

1. Section 2.1. Explain what α-ratio is.

2. P. 7, line 172: “temporal informatio” --> “information”

**********

what does this mean?). If published, this will include your full peer review and any attached files.

If you choose “no”, your identity will remain anonymous but your review may still be made public.

**Do you want your identity to be public for this peer review?**  For information about this choice, including consent withdrawal, please see our Privacy Policy

Reviewer #1: No

Reviewer #2: No

**********

[NOTE: If reviewer comments were submitted as an attachment file, they will be attached to this email and accessible via the submission site. Please log into your account, locate the manuscript record, and check for the action link "View Attachments". If this link does not appear, there are no attachment files.]

To ensure your figures meet our technical requirements, please review our figure guidelines: https://journals.plos.org/plosone/s/figures

You may also use PLOS’s free figure tool, NAAS, to help you prepare publication quality figures: https://journals.plos.org/plosone/s/figures#loc-tools-for-figure-preparation.

NAAS will assess whether your figures meet our technical requirements by comparing each figure against our figure specifications.

# Author comment: Author response to Decision Letter 2

**DOI:** 10.1371/journal.pone.0356236.r004

24 Jul 2026

please see our response letter

Attachment Submitted filename: LombardGainModel_PLOSONE_rebuttalLetter2(1).pdf

# Peer review: Decision Letter 2

**DOI:** 10.1371/journal.pone.0356236.r005

**Authors:** Jie Wang

2 Aug 2026

Spectro-temporal vs. Spectral Features to Predict the Lombard Gain in Mandarin Chinese

PONE-D-26-04256R2

Dear Dr. Scharf,

We’re pleased to inform you that your manuscript has been judged scientifically suitable for publication and will be formally accepted for publication once it meets all outstanding technical requirements.

Within one week, you’ll receive an e-mail detailing the required amendments. When these have been addressed, you’ll receive a formal acceptance letter and your manuscript will be scheduled for publication.

An invoice will be generated when your article is formally accepted. Please note, if your institution has a publishing partnership with PLOS and your article meets the relevant criteria, all or part of your publication costs will be covered. Please make sure your user information is up-to-date by logging into Editorial Manager at Editorial Manager® and clicking the ‘Update My Information' link at the top of the page. For questions related to billing, please contact billing support.

If your institution or institutions have a press office, please notify them about your upcoming paper to help maximize its impact. If they’ll be preparing press materials, please inform our press team as soon as possible -- no later than 48 hours after receiving the formal acceptance. Your manuscript will remain under strict press embargo until 2 pm Eastern Time on the date of publication. For more information, please contact onepress@plos.org.

Kind regards,

Jie Wang, Ph.D.

Academic Editor

PLOS One

Additional Editor Comments (optional):

Reviewers' comments:

Reviewer's Responses to Questions

**Comments to the Author**

Reviewer #1: All comments have been addressed

Reviewer #2: All comments have been addressed

**********

2. Is the manuscript technically sound, and do the data support the conclusions?

Reviewer #1: Yes

Reviewer #2: Yes

**********

3. Has the statistical analysis been performed appropriately and rigorously?

Reviewer #1: Yes

Reviewer #2: Yes

**********

4. Have the authors made all data underlying the findings in their manuscript fully available?

The PLOS Data policy

Reviewer #1: Yes

Reviewer #2: Yes

**********

5. Is the manuscript presented in an intelligible fashion and written in standard English?

Reviewer #1: Yes

Reviewer #2: Yes

**********

Reviewer #1: (No Response)

Reviewer #2: The authors have satisfactorily addressed my previous comments. I believe that the manuscript is now suitable for publication.

**********

what does this mean?). If published, this will include your full peer review and any attached files.

If you choose “no”, your identity will remain anonymous but your review may still be made public.

**Do you want your identity to be public for this peer review?**  For information about this choice, including consent withdrawal, please see our Privacy Policy

Reviewer #1: No

Reviewer #2: No

**********

# Editor report: Acceptance letter

**DOI:** 10.1371/journal.pone.0356236.r006

**Authors:** Jie Wang

PONE-D-26-04256R2

PLOS One

Dear Dr. Scharf,

I'm pleased to inform you that your manuscript has been deemed suitable for publication in PLOS One. Congratulations! Your manuscript is now being handed over to our production team.

At this stage, our production department will prepare your paper for publication. This includes ensuring the following:

* All references, tables, and figures are properly cited

* All relevant supporting information is included in the manuscript submission,

* There are no issues that prevent the paper from being properly typeset

You will receive further instructions from the production team, including instructions on how to review your proof when it is ready. Please keep in mind that we are working through a large volume of accepted articles, so please give us a few days to review your paper and let you know the next and final steps.

Lastly, if your institution or institutions have a press office, please let them know about your upcoming paper now to help maximize its impact. If they'll be preparing press materials, please inform our press team within the next 48 hours. Your manuscript will remain under strict press embargo until 2 pm Eastern Time on the date of publication. For more information, please contact onepress@plos.org.

You will receive an invoice from PLOS for your publication fee after your manuscript has reached the completed accept phase. If you receive an email requesting payment before acceptance or for any other service, this may be a phishing scheme. Learn how to identify phishing emails and protect your accounts at https://explore.plos.org/phishing.

If we can help with anything else, please email us at customercare@plos.org.

Thank you for submitting your work to PLOS ONE and supporting open access.

Kind regards,

PLOS ONE Editorial Office Staff

on behalf of

Dr. Jie Wang

Academic Editor

PLOS One
